# *Salmonella enterica* serovar Typhi and gallbladder cancer: a case–control study and meta‐analysis

**DOI:** 10.1002/cam4.915

**Published:** 2016-10-11

**Authors:** Jill Koshiol, Aniela Wozniak, Paz Cook, Christina Adaniel, Johanna Acevedo, Lorena Azócar, Ann W. Hsing, Juan C. Roa, Marcela F. Pasetti, Juan F. Miquel, Myron M. Levine, Catterina Ferreccio, the Gallbladder Cancer Chile Working Group, Carmen Gloria Aguayo, Sergio Baez, Alfonso Díaz, Héctor Molina, Carolina Miranda, Claudia Castillo, Andrea Tello, Gonzalo Durán, Carolina Paz Delgado, Rodrigo Quevedo, Susana Pineda, Tiare la Barra, Cristian Reyes, Cristina Alegría, Claudia Aguayo, Héctor Losada, Juan Carlos Arraya, Enrique Bellolio, Oscar Tapia, Jaime López, Karie Medina, Paulina Barraza, Sandra Catalán, Pía Riquelme, Lorena Órdenes, Raúl Garcés, Claudia Duarte, Allan Hildesheim

**Affiliations:** ^1^ Infections and Immunoepidemiology BranchDivision of Cancer Epidemiology and GeneticsNational Cancer InstituteMD; ^2^ Laboratory of MicrobiologyPontificia Universidad Católica de ChileSantiagoChile; ^3^ Escuela de MedicinaAdvanced Center for Chronic DiseasesACCDiSPontificia Universidad Católica de ChileSantiagoChile; ^4^ Department of GastroenterologySchool of MedicinePontificia Universidad Católica de ChileSantiagoChile; ^5^ Stanford Cancer InstitutePalo AltoCA; ^6^ Department of Health Research and PolicyStanford School of MedicinePalo AltoCA; ^7^ Department of PathologySchool of MedicinePontificia Universidad Católica de ChileSantiagoChile; ^8^ Center for Vaccine DevelopmentUniversity of Maryland School of MedicineBaltimoreMD

**Keywords:** Chile, epidemiology, gallbladder cancer, meta‐analysis, *Salmonella enterica* serovar Typhi, Vi antibodies

## Abstract

In Chile, where gallbladder cancer (GBC) rates are high and typhoid fever was endemic until the 1990s, we evaluated the association between *Salmonella enterica* serovar Typhi (*S. *Typhi) antibodies and GBC. We tested 39 GBC cases, 40 gallstone controls, and 39 population‐based controls for *S*. Typhi Vi antibodies and performed culture and quantitative polymerase chain reaction for the subset with bile, gallstone, tissue, and stool samples available. We calculated gender and education‐adjusted odds ratios (ORs) and 95% confidence intervals (CIs) for the association with GBC. We also conducted a meta‐analysis of >1000 GBC cases by combining our results with previous studies. GBC cases were more likely to have high Vi antibody titer levels than combined controls (OR: 4.0, 95% CI: 0.9–18.3), although *S. *Typhi was not recovered from bile, gallstone, tissue, or stool samples. In our meta‐analysis, the summary relative risk was 4.6 (95% CI: 3.1–6.8, *P*_heterogeneity_=0.6) for anti‐Vi and 5.0 (95% CI: 2.7–9.3, *P*_heterogeneity_ = 0.2) for bile or stool culture. Our results are consistent with the meta‐analysis. Despite differences in study methods (e.g., *S. *Typhi detection assay), most studies found a positive association between *S*. Typhi and GBC. However, the mechanism underlying this association requires further investigation

## Introduction

Gallbladder cancer (GBC) is rare, although the incidence varies greatly in different parts of the world. It is also highly lethal, with a 5‐year survival of approximately 12% 1, 2, 3, 4. Although gallstones are a major risk factor for GBC in high‐risk areas like Chile, presenting in >95% of GBC cases 5, it has been estimated that only 1% of gallstone patients will develop GBC 6.

Chronic biliary infection with *Salmonella enterica* serovar Typhi (*S. *Typhi), the causative agent of typhoid fever, has been proposed as one possible additional risk factor for GBC 2. This hypothesis developed from a case report of a woman who had documented typhoid fever for 30 years before hospitalization due to GBC, with *S. *Typhi subsequently isolated from bile and from the wall of the gallbladder 7. Several retrospective studies in the United States 8, Denmark 9, and Scotland 10 have provided strong evidence that chronic *S. *Typhi carriers have a significantly higher risk of death from GBC in comparison with the general population. More recently, several case–control studies have reported significant associations between *S. *Typhi and GBC, many of them coming from Northern India, where both typhoid fever and GBC are endemic. These associations are not always supported by evidence of *S. *Typhi from biologic specimens, but methods for detecting *S. *Typhi in the gallbladder may not be sufficiently sensitive, while Vi antibody serostatus may not reflect *S. *Typhi infection in the gallbladder itself. Thus, the nature of the association between *S. *Typhi and GBC remains unclear and is hindered by the limitations of the assays.

The hypothesis that *S. *Typhi may be linked to GBC is especially relevant in Chile, which has among the highest GBC incidence rates in the world (12.8 per 100,000 in women and 6.3 per 100,000 in men) 11, 12, 13. The annual country‐wide incidence of typhoid fever ranged from ~40 to 60 cases per 100,000 population between 1970 and 1976 and then rose abruptly from 1977 to 1984, with incidence rates ranging from >90 to 121 cases per 100,000 2, 14. Vaccination of large cohorts of schoolchildren between 1980 and 1985 with live oral typhoid vaccine lowered the annual rates in Santiago by 1990. Several sanitation interventions introduced following the 1991 outbreak of cholera lowered the annual rates dramatically beginning in 1992 to <20 cases per 100,000 and to <10 cases per 100,000 by 2000. Although *S. *Typhi exposure is now at low levels, exposure to *S*. Typhi when it was endemic decades ago could affect current trends in GBC rates if chronic carriage increases the risk of GBC. Given that GBC is the second leading cause of cancer death in Chilean women 11, 15, understanding the role of *S. *Typhi in gallbladder carcinogenesis is particularly relevant for early detection and prevention strategies.

We hypothesized that GBC cancer cases would have higher titers of IgG antibody to the Vi capsular polysaccharide of *S. *Typhi than controls with or without gallstones. We tested this hypothesis in 39 GBC cases, 40 gallstone controls, and 39 population‐based controls without gallstones, recruited from three public hospitals and the areas that they service in Chile. We also used culture and quantitative polymerase chain reaction (qPCR) in an attempt to detect *S*. Typhi in a subset of participants with bile, gallstone, gallbladder tissue, or stool samples available. In addition, we conducted a meta‐analysis to evaluate the effects of study design issues, such as differences in referent groups, assays, and geographical region.

## Materials and Methods

### Study population

As recently described 16, we enrolled incident GBC cases without prior cancer between April 2012 and August 2013 through rapid ascertainment in three public hospitals in Chile. We recruited age‐ and sex‐matched gallstone controls who had cholecystectomy in the same week as the case and age‐ and sex‐matched population‐based controls identified from the registry of beneficiaries at the local health center or through neighborhood sampling. Eighty‐four percent of GBC cases (49/58), 88% of gallstone controls (37/42), and 60% of population‐based controls consented to participate (49/82). We included all participants with serum available. Eight population‐based controls had ultrasound‐detected gallstones. These individuals were recoded as gallstone controls. We also excluded excluded two population‐based controls with unknown gallstone status. Therefore, the current analysis included 39 GBC cases, 40 gallstone controls. Based on a previous meta‐analysis that provided a summary OR of 3.5 for antibody‐based *S. *Typhi detection and GBC 17, we estimated that with 39 GBC cases and two controls per case (gallstone and population‐based controls combined), we would have more than 80% power to confirm this association in our study population. The study was approved by institutional review boards of the US National Cancer Institute, Pontificia Universidad Católica, the Chilean Ministry of Health, and each hospital that contributed to the study as required. All participants provided written consent. Biospecimens were stored at −80°C.

### Laboratory testing

#### Elevated titers of serum antibody against Vi capsular polysaccharide

**Figure 1:**
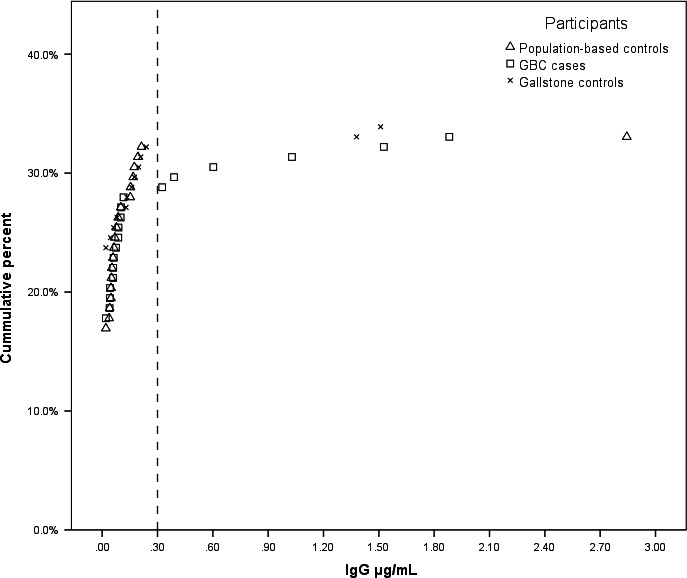
Distribution of detectable Vi antibody levels among gallbladder cancer cases, gallstone controls, and population‐based controls.

Serum samples were blindly tested for IgG antibodies to *S. *Typhi Vi capsular polysaccharide in the Applied Immunology Section of the Center for Vaccine Development, University of Maryland School of Medicine. Anti‐Vi antibody titers were measured by ELISA with modifications of the methods of Losonsky et al. 18 and Wahid et al. 19. We used biotinylated Vi (kindly provided by Dr. Andrew Lees). In addition, we ran a human Vi antibody standard 20 that allowed us to convert ELISA unit titers to *μ*g/mL of IgG Vi antibody. The lower limit of detection was 0.04 *μ*g/mL. Based on the distribution of Vi antibody titers among cases and controls (Fig. 1), we used ≥0.3 *μ*g/mL as the cut off for high Vi antibody titer levels. We also conducted sensitivity analyses using cut offs of 0.2 (the upper limit for seropositivity considering an assay variation of up to 25%), 0.5, 1, and 1.5 *μ*g/mL. We included duplicate aliquots for four individuals. The within subject coefficient of variation for these subjects was 5%.

#### Microbial assays

We tested for evidence of bacteria in tissue (*N* = 35), gallstones (*N* = 35), bile (*N* = 10), and stools (*N* = 35) from the subset of participants who had sufficient material available. Microbial assays were performed in the Laboratory of Microbiology at Pontificia Universidad Católica de Chile as described below.

### Culture and DNA extraction

#### Stool

Thirty‐five samples from 13 GBC cases, nine gallstone controls, and 13 population‐based controls were enriched in selenite and ox bile broth (Oxoid) and plated in Hektoen agar. The bacterial species among the suspicious colonies were identified by matrix‐assisted laser desorption/ionization time‐of‐flight mass spectrometry (MALDI‐TOF). Selenite broth cultures were centrifuged and DNA was extracted with the QIAamp DNA Stool Mini kit.

#### Gallbladder tissue, bile, and gallstones

A subset of participants had gallbladder tissue (10 GBC cases, 25 gallstone controls), bile (three GBC cases, seven gallstone controls), and gallstones (eight GBC cases, 28 gallstone controls) collected aseptically. Gallbladder tissue, bile, or gallstones were enriched in selenite and Tryptic soy broth. If the tissue sample was large, it was cut into smaller pieces prior to culture. One gallstone was cultured per participant. One mL of bile was cultured per participant. After overnight incubation, one aliquot of broth was used for culture, and one was used for DNA extraction. Cultures exhibiting visible growth were plated on blood and chocolate agar, and colonies that grew were identified by MALDI‐TOF. DNA was extracted using the QIAamp DNA Mini Kit according to the manufacturer's instructions. For DNA extraction from tissue, cultured tissue samples were homogenized, and 25 mg of tissue was combined with 1 mL of the culture broth and centrifuged at 1200 g for 2 min. DNA was extracted from the pellet. Supernatant was discarded and 180 *μ*L of lysis buffer and 20 *μ*L of proteinase K were added to the pellet. After two washing steps, DNA was eluted in 50 *μ*L of elution buffer. For DNA extraction from gallstones, cultured gallstones were sonicated in the culture medium for 1 min at 50 kHz, and 1 mL of culture broth was centrifuged for 2 min at 1200 g. DNA extraction continued as described above. For DNA extraction from bile, 1 mL of cultured bile was centrifuged for 2 min at 1200 g. DNA extraction continued as described above.

#### Detection of specific pathogens by PCR

All samples (bile, tissue, stones, stool) were analyzed by real‐time PCR (qPCR) to detect *S*. Typhi, employing primers to amplify *fliC,* which encodes the *S*. Typhi Phase 1 flagellin subunit (Hd) 21. The analytical sensitivity of the PCR was 14.5 *S*. Typhi genomes per qPCR reaction (data not shown). Conversely, DNA extracted from clinical isolates of *S*. Typhimurium, *S*. Choleraesuis, *S*. Paratyphi A, or *S*. Enteritidis did not amplify. Gel‐based PCR was used to detect *Salmonella* species, utilizing primers that amplify *invA* encoding a *Salmonella* Pathogenicity Island 1 protein required for invasion of epithelial cells 22). PCR products for *Salmonella* species were visualized on agarose gels, 1.5%. DNA extracted from a clinical isolate of *S*. Typhi was used as the positive control in qPCR and gel‐based PCR tests. PCR‐grade water was used as the negative control in all tests. Human RNAse P gene was used as the internal quality control to ensure proper DNA extraction and the absence of PCR inhibitors 23, 24.

### Statistical analysis

We compared participants using the Kruskal–Wallis test for difference in median or nonzero correlation chi‐square test for categorical comparisons (Table 1). Conditional and unconditional logistic regression models produced similar results for the association of *S. *Typhi with GBC compared to gallstone controls and population‐based controls; thus, we used unconditional logistic regression to calculate odds ratios (ORs) and 95% confidence intervals (95% CIs). We evaluated the association between high titers of anti‐Vi antibody seropositivity (≥0.3 *μ*g/mL for the primary analysis) and GBC compared gallstone controls and population‐based controls combined using standard logistic regression. In addition, we compared GBC to gallstone controls and population‐based controls separately using polytomous logistic regression. We also explored if high Vi antibody titer levels were associated with gallstones by comparing gallstone controls to population‐based controls without gallstones. Using forward modeling, we assessed potential confounders including continuous age, gender, study site (Concepcion and Temuco, which have high risk of GBC, vs. Santiago), self‐reported Mapuche ancestry, BMI (based on self‐report of weight more than 3 years ago or average adult weight and categorized according to the WHO guidelines: BMI <18.50 = underweight, 18.50–24.99 = normal weight, 25.00–29.99 = overweight, ≥30 = obese), education (ordinal categories of less than 6 years of education, 7–9 years, 10–12 years, and 13 years of more), diabetes, smoking (ever vs. never), family history of GBC. Gender and education changed the ORs by more than 10% and were therefore retained in the adjusted models. All analyses were conducted using SAS 9.3 (SAS Institute Inc., Cary, NC).

**Table 1:** Characteristics of gallbladder cancer cases (GBC), gallstone patients, and population‐based controls tested for *Salmonella* Typhi Vi antibody seropositivity

	GBC(*N* = 39)	Gallstone controls(*N* = 40)	Population‐based controls(*N* = 39)	*P*‐value^b^
Age, median (range)	62 (41–77)	60 (40–79)	66 (37–75)	0.9
Male gender, *N*(%)^a^	7 (17.9)	7 (18.4)	8 (20.5)	0.8
High‐risk site (Concepcion or Temuco), N(%)	20 (51.3)	18 (45.0)	15 (38.5)	0.3
Self‐reported Mapuche ancestry, *N*(%)^a^	4 (10.3)	2 (5.4)	2 (5.1)	0.4
Education level, *N*(%)^a^				
≤6 years	21 (53.8)	17 (44.7)	12 (30.8)	0.004
7–9 years	9 (23.1)	8 (21.1)	5 (12.8)	
10–12 years	6 (15.4)	9 (23.7)	13 (33.3)	
≥13 years	3 (7.7)	4 (10.5)	9 (23.1)	
Body mass index weight category, N(%)^a^^,^ c				0.7
Normal weight	14 (41.2)	8 (25.0)	12 (33.3)	
Overweight	12 (35.3)	15 (46.9)	16 (44.4)	
Obese	8 (23.5)	9 (28.1)	8 (22.2)	
Diabetes, N(%)^a^	10 (25.6)	6 (16.2)	6 (15.4)	0.3
Smokers, N(%)^a^	12 (30.8)	14 (37.8)	21 (53.8)	0.04
Family history of GBC, N(%)^a^	2 (7.1)	0 (0.0)	1 (3.6)	0.5
Self‐reported typhoid fever, N(%)^a^	3 (8.1)	2 (5.7)	3 (7.7)	0.9
Elevated Vi antibody seropositivity, N(%)^d^	6 (15.4)	2 (5)	1 (2.6)	0.03

a Percentages exclude individuals with missing data.

b Kruskal–Wallis test for difference in median or nonzero correlation chi‐squared test for categorical comparisons.

c Based on self‐report of weight more than 3 years ago or average adult weight.

d A titer ≥0.3ug/mL was considered as having elevated Vi antibody titers.

### Literature review and meta‐analysis

We searched for published studies on *Salmonella* and GBC in MEDLINE (via PubMed) through 10 February 2016 using the terms (“hepatobiliary cancer” OR “hepatopancreatobiliary cancer” OR “biliary tract cancer” OR “biliary tract carcinoma” OR “bile duct cancer” OR “bile duct carcinoma” OR “gallbladder cancer” OR “gall bladder cancer” OR “gallbladder carcinoma” OR “gall bladder carcinoma”) AND (“Salmonella Typhi” OR Salmonella OR “typhoid fever” OR “S. typhi” OR “S typhi” OR “S. Typhi” OR “S Typhi” OR “S. paratyphi” OR “S paratyphi” OR “S. Paratyphi” OR “S Paratyphi”). No restrictions were placed on language or publication starting date. Peer‐reviewed publications that evaluated *Salmonella* and GBC were eligible if they either reported or had calculable relative risks (risk ratios, rate ratios, ORs, or standardized incidence or mortality rates, hereafter termed ‘‘relative risks’’ and referred to RRs) and corresponding 95% confidence intervals (CIs) for the association between *Salmonella* and GBC.

We abstracted RRs and 95% CIs if they were reported, or calculated them ourselves for the association between *Salmonella* and GBC. For author‐calculated RRs, 0.5 was added to each of the four interior cells if one of the cells contained zero. Abstracted data included *Salmonella* detection method (culture, antibodies against somatic antigens (TO) or flagellar antigens (TH), antibodies against VI antigen, nested PCR for the *S*. Typhi flagellin subunit gene, self‐report, physician‐diagnosed, *S*. Typhi lypopolysaccharide), biospecimen if applicable (stool, bile, serum, gallstones, tissue), outcome (prevalent GBC, incident GBC, GBC mortality, hepatobiliary cancer mortality, hepatobiliary cancer incidence), and other study characteristics (e.g., sample size, study design, geographic location). For publications based on the same study population 10, 25, 26, 27, 28, 29, 30, 31, 32, 33, 34, 35, we used the article with the largest number of participants unless multiple articles could contribute to separate analyses. We initially extracted data on two studies that we later excluded from analyses because they had ecological 36 or case series 34 study designs.

We evaluated the association between *Salmonella* and GBC using stratified random‐effects meta‐analysis and examined key study characteristics and variation across studies using restricted maximum likelihood metaregression. Some studies provided multiple RRs with differing *Salmonella* detection methods or outcome referent groups. In these cases, we applied the following decision rules to select one RR per study for any given analysis: (1) if crude and adjusted estimates available, chose adjusted estimate; (2) choose results with the largest number of cases, then the largest number of controls; if the number of cases is similar and the number of controls very different, base choice on the largest number of controls; (3) if there are multiple results with the same number of cases and controls but different *S. *Typhi assays, choose the more specific assay (culture, DNA, Vi antibody seropositivity, then Widal). We used Cochran's Q two‐sided homogeneity *P*‐value 37 to evaluate heterogeneity in RRs and funnel plots to assess asymmetry, which can reflect publication bias, random error, or study characteristics associated with sample size 38, using Begg rank correlation 39 and Egger regression 40.

## Results

The current analysis included 39 GBC cases, 40 gallstone controls, and 39 population‐based controls with serum available. The sociodemographic characteristics were generally similar across cases and controls, although GBC cases were less likely to have higher education (*χ* ^2^ *P* = 0.004) or to be smokers (*χ* ^2^ *P* = 0.04) (Table 1). In addition, 15.4% of GBC cases had high Vi antibody levels (≥0.3 *μ*g/mL) compared to 5.0% of gallstone controls and 2.6% of population‐based controls.

High Vi antibody titer levels were associated with an increased risk of GBC compared to all controls combined using standard logistic regression [OR (95% CI): 4.6 (1.1–19.5)], and the association remained elevated after adjustment for gender and education [4.0 (0.9–18.3)]. GBC cases were more likely to have high Vi antibody titer levels than combined controls even when higher or lower cut offs were used to define high Vi antibody titer level (Table S1). Comparing to gallstone and population‐based controls separately using polytomous logistic regression, the gender‐ and education‐adjusted ORs for elevated Vi antibody seropositivity and GBC were 3.1 (0.6–17.7) versus gallstone controls, and 5.6 (0.6–52.8) versus population‐based controls. Gallstone controls were about two times as likely to be Vi antibody seropositive compared to population‐based controls [1.8 (0.1–21.9)], although the estimates were imprecise.

None of the stool specimens analyzed had Salmonella Typhi or non‐Typhi *Salmonella*. One GBC case and one gallstone control were positive for non‐Typhi *Salmonella* in tissue and bile specimens, respectively, but none had evidence of *S*. Typhi (Table S2).

**Figure 2:**
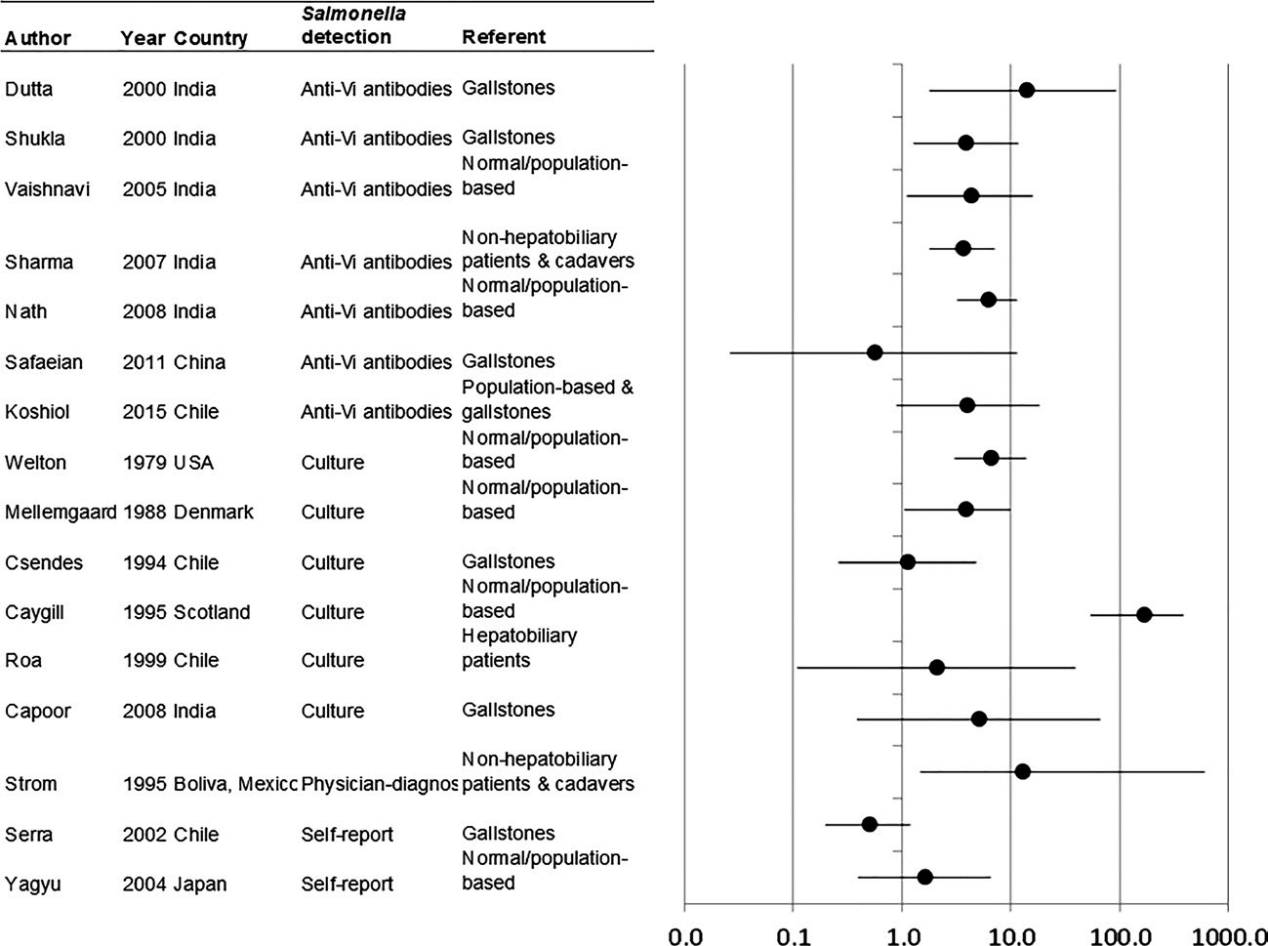
Relative risks (RRs) and 95% confidence intervals (CIs) for associations between *Salmonella* and gallbladder cancer (GBC) in the published literature.

The meta‐analysis included data from 22 published studies on *Salmonella* and GBC, along with this study (Table 2). Of these 22 studies, 18 (82%) were case–control studies 8, 26, 27, 28, 29, 30, 31, 32, 33, 35, 41, 42, 43, 44, 45, 46, 47, 48 and four (18%) were cohort studies 9, 10, 25, 49. Most studies were conducted in Asia (*N* = 13, 59%), followed by Central/Southern America (*N* = 5, 23%), Europe (*N* = 3, 14%), and the United States (*N* = 1, 5%). Eleven of these studies overlapped with other studies from the same study population. After conducting an initial overall meta‐analysis based on results from 16 studies with independent study populations including 1109 cases (Fig. 2), we found that the estimates were highly heterogenous (P_heterogeneity_<0.001). The funnel plot appeared generally symmetrical, and there was no evidence of bias using either the Begg or Egger methods (*P* = 0.9 and 0.7, respectively). However, two studies lay outside the funnel: Caygill 1995 [(standardized mortality ratio: 167 (54–391)] and Serra 2002 [OR: 0.5 (0.2–1.2)]. After removing these two studies, the remaining 14 studies did not appear heterogeneous (*P*_heterogeneity_ = 0.5) and produced a summary RR of 4.3 (3.2–5.8). We excluded the two outlying studies from further analyses.

**Table 2:** Studies of *Salmonella* and gallbladder cancer (GBC)

Reference	Country	Study design	Population	Specimen	*S*. Typhi detection	Outcome	Referent	*N* cases	*N* noncases	% GBC *S*. Typhi+	% noncase *S*. Typhi+	RR (95% CI)	Adjusted	Hand‐calculated
Welton 1979 8	USA	Case–control	General	Stool	Culture	Hepatobiliary cancer mortality	Individuals from general population who died	37	1376	74.3%	32.2%	6.6 (3.1–14.0)	No	Yes
Mellemgaard 1988 9	Denmark	Cohort	General	Stool	Culture	Hepatobiliary cancer incidence	Denmark age, time, and sex‐specific incidence rates	NS	NS			3.9 (1.1–9.9)	Yes	No
Caygill 1994 10	UK	Cohort	General	Stool	Culture	GBC mortality	Morality rates	83	NS	6.0%		167 (54.1–389)	Yes	No
				Stool	Culture		Acute typhoid	83	386	6.0%		52.3 (3.0–989.5)	No	Yes^a^
Csendes 1994 41	Chile	Case–control	Hospital	Bile	Culture	Incident GBC	Gallstone patients	47	52	8.5%	7.7%	1.1 (0.3–4.7)	No	Yes
				Bile	Culture		Acute cholecystitis	47	19		26.3%	0.3 (0.1–1.1)	No	Yes
				Bile	Culture		Common bile duct stones	47	39		10.3%	0.8 (0.2–3.5)	No	Yes
Caygill 1995 25	UK	Cohort	General	Stool	Culture	Hepatobiliary cancer mortality	Morality rates	167	NS	3.0%		167 (54–391)	Yes	No
				Stool	Culture	GBC mortality	Acute typhoid	167	507	3.0%		57.3 (3.1–1043.9)	No	Yes^a^
Capoor 2008 45	Bolivia, Mexico	Case–control	Hospital	NA	MD‐diagnosed typhoid	Prevalent GBC	Abdominal surgery patients without biliary cancer or stones	84	126			12.7 (1.5–598)	Yes	No
				Serum	*S*. Typhi LPS		Abdominal surgery patients without biliary cancer or stones	15	8	46.7%	50.0%	0.9 (0.2–4.9)	No	Yes
				Serum	*S. Typhi LPS*		Gallstone/bile duct stone patients	15	10	46.7%	50.0%	0.9 (0.2–4.3)	No	Yes
Singh 1996 30	India	Case–control	Hospital	Bile	Culture	Prevalent GBC	Gallstone patients	38	67	13.2%	3.0%	4.9 (0.9–26.8)	No	Yes
Nath 1997 27	India	Case–control	Hospital	Bile	Culture	Prevalent GBC	Patients with stones or no biliary pathology	28	73	14.3%	1.4%	12 (1.3–112.7)	No	Yes
				Bile	Culture		Stones only	28	56	14.3%	1.8%	9.2 (0.97–86.4)	No	Yes
				Bile	Culture		No biliary path only	28	17	16.1%	2.9%	6.7 (0.3–132.8)	No	Yes^a^
Roa 1999 62	Chile	Case–control	Hospital	Bile	Culture	Prevalent GBC or dysplasia	Chronic and acute cholecystitis	29	579	1.7%	0.8%	2.1 (0.1–39.4)	No	Yes^a^
Dutta 2000 43	India	Case–control	Hospital	Serum	VI (ELISA)	Incident GBC	Gallstone patients	37	80	16.2%	2.5%	14 (1.8–92)	Yes	No
Shukla 2000 29	India	Case–control	Hospital	Serum	VI (IHA)	Prevalent GBC	Gallstone patients	51	56	29.4%	10.7%	3.9 (1.3–11.7)	Yes	No
				Serum	VI (IHA)		No hepatobiliary disease	51	40	29.4%	5.0%	7.2 (2.2–23.4)	Yes	No
				Serum	Widal test		Gallstone patients	51	56	39.2%	42.9%	0.9 (0.4–1.9)	No	Yes
				Serum	Widal test		No hepatobiliary disease	51	40	39.2%	40.0%	0.97 (0.4–2.3)	No	Yes
Serra 2002 42	Chile	Case–control	Hospital	NA	Self‐report	Prevalent GBC	Gallstone patients	114	114	9.6%	7.9%	0.5 (0.2–1.2)	Yes	No
Pandey 2003 28	India	Case–control	Hospital	NA	Self‐report	Incident GBC	Gallstone patients	64	101	21.9%	12.9%	1.3 (0.9–2.0)	No	Yes
Hazrah 2004 26	India	Case–control	Hospital	Gallstones	Culture	Incident GBC	83 chronic cholecystitis, 2 empyema, 1 periampullary cancer	14	86	3.6%	2.9%	1.2 (0.1–25.5)	No	Yes^a^
Yagyu 2004 49	Japan	Cohort	General	NA	Self‐report	GBC mortality	Noncases	89	91895	2.2%	1.4%	1.6 (0.4–6.5)	No	Yes
			Males only	NA	Self‐report		Male noncases	34	38603	5.9%	1.6%	2.1 (0.5–8.7)	Yes	No
Vaishnavi 2005 33	India	Case‐control	Hospital	Serum	VI (ELISA)	Prevalent GBC	Gallbladder/bile duct stones	27	196	7.4%	10.2%	0.7 (0.2–2.8)	No	Yes
				Serum	VI (ELISA)		Cholangiocarcinoma, obstructive jaundice, or carcinoma of pancreas/ampulla	27	33	7.4%	12.1%	0.6 (0.2–1.6)	No	Yes
				Serum	VI (ELISA)		Miscellaneous gastrointestinal ailments	27	112	7.4%	9.8%	0.7 (0.2–2.7)	No	Yes
				Serum	VI (ELISA)		Miscellaneous diseases	27	78	7.4%	9.0%	0.8 (0.2–2.7)	No	Yes
				Serum	VI (ELISA)		Consecutive healthy blood donors	27	705	7.4%	1.8%	4.3 (1.1–16.1)	No	Yes
Sharma 2007 32	India	Case–control	Hospital	Serum	TO	Prevalent GBC	Gallstone patients	65	125	40.0%	62.4%	0.4 (0.2–0.7)	No	Yes
				Serum	TH		Gallstone patients	65	125	30.8%	9.6%	4.2 (1.9–9.3)	No	Yes
				Serum	TO		Laparotomy for diseases other than hepatobiliary tract	65	200	40.0%	9.0%	6.7 (3.4–13.5)	No	Yes
				Serum	TH		Laparotomy for diseases other than hepatobiliary tract	65	200	30.8%	11.0%	3.6 (1.8–7.2)	No	Yes
				Serum	VI (IHA)		Gallstone patients	65	125	30.8%	9.6%	4.2 (1.9–9.3)	No	Yes
				Serum	VI (IHA)		Laparotomy for diseases other than hepatobiliary tract	65	200	30.8%	11.0%	3.6 (1.8–7.2)	No	Yes
				Bile	Culture		Gallstone patients	65	125	24.6%	3.2%	9.9 (3.1–31.0)	No	Yes
Capoor 2008 46	India	Case–control	Hospital	Tissue, gallstones, bile	Culture	Prevalent GBC	Cholelithiasis + acute cholecystitis	6	53	16.7%	3.8%	5.1 (0.4–66.7)	No	Yes
Nath 2008 31	India	Case–control	Hospital	Serum	VI (IHA)	Prevalent GBC	Other gallbladder diseases	52	223	38.5%	13.9%	3.9 (2.0–7.6)	No	Yes
				Serum	VI (IHA)		Healthy adults	52	508	38.5%	9.3%	6.1 (3.3–11.6)	No	Yes
				Serum	TO (Widal test)		Other gallbladder diseases	52	223	23.1%	11.7%	2.3 (1.1–4.9)	No	Yes
				Serum	TO (Widal test)		Healthy adults	52	508	23.1%	13.4%	1.9 (0.97–3.9)	No	Yes
				Serum	TH (Widal test)		Other gallbladder diseases	52	223	21.2%	3.6%	7.2 (2.7–19.0)	No	Yes
				Serum	TH (Widal test)		Healthy adults	52	508	21.2%	11.8%	2.0 (0.98–4.1)	No	Yes
				Bile	Culture		Other gallbladder diseases	52	223	3.8%	0.9%	4.5 (0.6–32.4)	No	Yes
				Bile	Culture		Cadavers without gallbladder pathology	52	424	3.8%	0.1%	42.0 (2.0–887.8)	No	Yes^a^
				Bile, stone, tissue, blood	PCR + flagellin sequencing		Other gallbladder diseases	52	223	67.3%	42.6%	2.8 (1.5–5.2)	No	Yes
				Bile, stone, tissue, blood	PCR + flagellin sequencing		Cadavers without gallbladder pathology	52	424	67.3%	8.3%	11.1 (6.5–22.0)	No	Yes
Tewari 2010 35	India	Case–control	Hospital	Serum	TO and TH (Widal)	Prevalent GBC	Gallstone patients	54	54	44.4%	24.1%	2.5 (1.1–5.7)	No	Yes
				Serum	VI (IHA)		Gallstone patients	54	54	22.2%	9.3%	2.8 (0.9–8.6)	No	Yes
				Tissue	Culture		Gallstone patients	54	54	6.5%	0.9%	7.7 (0.4–153.0)	No	Yes^a^
				Bile	PCR (*flagellin*)		Gallstone patients	54	54	4.6%	0.9%	5.2 (0.2–110.7)	No	Yes^a^
				Tissue	PCR (*flagellin*)		Gallstone patients	54	54	34.3%	0.9%	55.2 (3.2–945.8)	No	Yes^a^
Safaeian 2011 47	China	Case–control (population‐based)	General	Serum	VI (ELISA)	Incident GBC	Gallstone patients	262	728	0.2%	0.3%	0.6 (0.03–11.6)	No	Yes^a^
Koshiol 2016	Chile	Case–control (population‐based)	General	Serum	VI (ELISA)	Incident GBC	Gallstone patients	39	40	15.4%	5.0%	3.1 (0.6–17.7)	Yes	No
				Serum	VI (ELISA)		Population‐based controls	39	39	15.4%	2.6%	5.6 (0.6–52.8)	Yes	No
				Serum	VI (ELISA)		Gallstone patients + population‐based controls	39	79	15.4%	3.8%	4.0 (0.9–18.3)	Yes	No

LCL, lower confidence limit; NA, not applicable; NS, not‐specified; TH, antibodies to flagellar Salmonella antigen; TO, antibodies to somatic Salmonella antigen; UCL, upper confidence limit; VI, antibodies to Vi *Salmonella* antibody.

a Added 0.5 to 0 cells.

Studies of Vi antibody seropositivity and bile culture produced similar results [summary RR (95% CI): 4.6 (3.1–6.8) and 4.7 (1.5–14.6)] (Table 3). Stool culture produced slightly higher [summary RR 5.5 (3.0–10.4)] but not substantially different [ratio of RRs: 1.2 (0.6–2.5)] estimates than Vi antibody‐based estimates. Combining bile culture and stool culture‐based estimates, the summary RR was 5.0 (2.7–9.3, *P*_heterogeneity_ = 0.2). Estimates based on self‐report were much lower than Vi antibody‐based estimates [(ratio of RRs: 0.3 (0.2–0.5)]. Results did not vary greatly by additional study characteristics or exhibit notable heterogeneity with the possible exception of outcome referent group (Table 3). Estimates comparing GBC to controls from the general population [summary RR (95% CI): 5.1 (3.4–7.6)] or nonhepatobiliary patients and cadavers [5.5 (2.2–13.9)] tended to be stronger than those comparing to gallstones [2.5 (1.4–4.2)], although estimates using nonhepatobiliary patients or cadavers as referent were also more heterogeneous (*P*_heterogeneity_=0.003 vs. 0.2).

**Table 3:** Effect of study characteristics on the association between *Salmonella* and gallbladder cancer (GBC) in a meta‐analysis of the published literature

			Summary effect estimate^a^		Ratio of effect estimates	
Study characteristic	N studies	Cochrane's Q *P*‐value	RR	95% CI	Ratio of RRs	95% CI
*Salmonella* detection method						
VI antibody seropositivity	7	0.6	4.6	3.1–6.8	1.0	
Bile culture	5	0.1	4.7	1.5–14.6	1.0	0.5–2.4
Stool culture	2	0.4	5.5	3.0–10.4	1.2	0.6–2.5
Self‐report	2	0.8	1.3	0.9–2.0	0.3	0.2–0.5
Referent group						
Stones (gallstones and/or bile duct)	10	0.2	2.5	1.4–4.2	1.0	
General population^b^	6	0.6	5.1	3.4–7.6	1.9	0.7–4.8
Hepatobiliary patients	4	0.005	2.1	0.4–11.0	0.9	0.3–2.9
Nonhepatobiliary patients and cadavers	6	0.003	5.5	2.2–13.9	2.2	0.8–5.9
Study design						
Case–control	12	0.6	4.6	3.3–6.4	1.0	
Cohort	2	0.3	2.7	1.1–6.6	0.6	0.2–1.5
Region						
Asia	8	0.5	4.3	3.0–6.3	1.0	
Central/South America	4	0.4	2.5	1.0–6.3	0.6	0.2–1.6
Population						
Hospital	9	0.5	4.4	3.0–6.4	1.0	
General	5	0.3	3.9	2.1–7.2	1.0	0.5–1.8
Statistical analysis						
Crude/hand‐calculated	8	0.3	4.3	2.9–6.5	1.0	
Adjusted	6	0.6	3.9	2.2–7.0	0.9	0.4–1.7

a Random effects relative risks (RRs), 95% confidence limits (95% CIs).

b Normal patients, population‐based controls, mortality rates, incidence rates.

## Discussion

In Chile, which has among the highest GBC incidence and mortality rates worldwide, we observed a trend toward a higher prevalence of elevated Vi antibody titers among GBC cases compared to gallstone and population‐based controls. Although the ORs were borderline significant, the magnitudes were high, with a fourfold increase for GBC cases compared to combined gallstone and population‐based controls and a 3.1‐fold and 5.6‐fold increase, respectively, for GBC cases compared separately to gallstone controls and population‐based controls.

These magnitudes are comparable to those from other studies. Our adjusted OR of 4.0 (0.9–18.3) for high‐titer Vi antibody seropositivity and GBC was similar to the meta‐analysis summary RR for Vi antibody seropositivity [(summary RR: 4.6 (3.1–6.8)]. In the meta‐analysis, associations between *S. *Typhi and GBC remained even when stratified by factors like *S. *Typhi detection method, outcome referent group, study design, geographical region, source of study population, and statistical adjustment. This consistency is striking.

However, the magnitude of the estimates appeared to be affected by the *S. *Typhi exposure assessment and outcome referent group used. Estimates based on self‐report produced a summary RR of 1.3 (0.9–2.0), likely reflecting poor measurement of exposure to *S. *Typhi since most people do not know that they have been exposed. With regard to outcome referent group, in our study, we combined gallstone and population‐based controls to gain precision since both referent groups produced strong ORs separately. The magnitude of the OR was stronger for GBC compared to population‐based controls than compared to gallstone controls, however. Similarly, in the meta‐analysis, general population controls and nonhepatobiliary patients/cadavers tended to produce stronger RRs than estimates compared to gallstone controls. *S. *Typhi is associated with gallstones. It is believed that this association is due to *S. *Typhi's propensity to form biofilms on gallstones 50 and to colonize abnormal gallbladder mucosa. However, potential reverse causality cannot be ruled out. Additional research is needed to tease apart the exact role of *S. *Typhi across the natural history of GBC carcinogenesis.

This need is highlighted by the fact that on an individual study level, the association with GBC is not always consistent for *S. *Typhi detected in samples from the gallbladder or gastrointestinal tract. For example, a previous study conducted in Santiago, Chile, when typhoid fever incidence was still high, found a similar prevalence of *S. *Typhi in bile cultures from GBC cases (8.5%) and gallstones controls (7.7%) and thus no association between *S. *Typhi and GBC [OR: 1.12 (0.26–4.74)] 51. A systematic study of bile specimens from cholecystectomies performed in Chile in the early 1980′s found *S. *Typhi or *S. *Paratyphi in 7.3% (73/1000) of bile cultures 52, but this prevalence may have included some transient *S. *Typhi or *S. *Paratyphi infections (i.e., infections that are detected in the first 12 months after recovery from acute typhoid fever or after subclinical infection), as well as chronic infections. A similar situation may be occurring in India today, where typhoid fever incidence rates in some areas are currently higher than those of Chile in the 1980′s and *S. *Typhi isolation in bile samples from GBC patients ranges from 14.3% 27 to 25% 32. Chile, in contrast, no longer has a high *S. *Typhi incidence rate. In our study, we did not succeed in culturing *S. *Typhi or in identifying *S. *Typhi DNA in bile, gallstones, gallbladder tissue, or stool samples, which could reflect reduced exposure more than 20 years after the control of typhoid hyperendemicity, or it may be due to lack of power given the small number of bile, gallstone, and tissue samples. Alternatively, the typhoid bacilli may reside in bile ducts rather than in the gall bladder *per se* in some chronic carriers. In any case, these findings highlight the value of the Vi antibody assay in population studies.

We did not have the ability to evaluate serotypes other than S. Typhi in the current study. *Salmonella* Paratyphi A and B do not produce Vi polysaccharide. *S*. Paratyphi C, expresses Vi, but it is a very rare cause of enteric fever 53 thus, the Vi antibody assay essentially only detects *S*. Typhi. Nevertheless, it is important to note that chronic *S*. Paratyphi A and *S*. Paratyphi B carriage also appear to contribute to gallbladder carcinogenesis. A previous study in Chile isolated *S. *Paratyphi A in 1.5% of 1000 bile samples and *S. *Paratyphi B in 8.8%, accounting for 6.8% and 41.1% of all *Salmonella* species isolated 52. In addition, Caygill et al. reported that their cohort of chronic *Salmonella* carriers included 61 *S. *Paratyphi A, B, or C carriers, 21 *S. *Typhi carriers, and one carrier with both *S*. Paratyphi and *S. *Typhi, although they did not describe the serotypes of the five GBC cases that developed among these carriers 10. Singh et al. reported one *S*. Paratyphi carrier and four *S*. Typhi carriers out of 38 GBC cases (2.6% and 10.5%, respectively) 30. Finally Nath et al. reported that 3.5% of 28 GBC cases had *S*. Paratyphi A and 10.7% had *S*. Typhi, while the corresponding figures were 0% and 1.8% among the 56 gallstone controls 27. These findings are intriguing, although information about serotypes other than *S*. Typhi are quite limited and require larger studies targeted toward these serotypes to draw conclusions.

In addition to the epidemiological evidence, several lines of biological evidence suggest that *S. *Typhi may contribute to gallbladder carcinogenesis. The capsular polysaccharide expressed by *S*. Typhi has been shown to suppress the inflammatory response of intestinal mucosa 54, 55, 56, while non‐typhoidal *Salmonella* serovariants such as *S*. Typhimurium and *S*. Enteritidis cause strong inflammatory responses in intestinal mucosa, including an influx of polymorphonuclear neutrophils. ViaB genetic mutants of *S*. Typhi that do not express Vi polysaccharide cause significantly more mucosal inflammation in a bovine intestinal loop model than wild‐type *S. *Typhi 55. If *S. *Typhi suppresses immune response in the gallbladder mucosa as well, it may create a chronic, low‐level inflammatory or regulatory immune environment that enhances the propensity to develop carcinogenic changes of the mucosa. *Salmonella*, especially *S. *Typhi 57, is well known to form biofilms upon contact with cholesterol gallstones and similar substrates, and the genes it expresses vary depending on whether it is in a biofilm state 50, 58. *S. *Typhi produces a number of carcinogens, including bacterial glucoronidase, secondary bile acids, nitroso compounds, and cytolethal distending toxin (CDT), a genotoxin with immunomodulatory capability that causes DNA damage in the nucleus of the infected cell 59. DNA damage may also result from increased free radical concentration due to chronic *S. *Typhi infection 60. Furthermore, a recent study found that *S. *Typhi could induce transformation in murine gallbladder organoids and fibroblasts, although only in the presence of predisposing mutations (i.e., inactivated *TP53* mutations and *c‐MYC* amplification), and that by causing such mutations in gallbladder tissue, *S. *Typhi may have stable transformative effects that remain even after the infection is eradicated 61. However, such mechanistic studies might best be done with *Salmonella* grown in biofilms that mimic the way they exist in the colonized gallbladder.

This study has weaknesses, of which sample size and lack of bile, tissue, and stool specimens in a proportion of participants are the most obvious. A future, larger study in Chile would address this issue. In addition, as a case–control study, we cannot rule out the potential for reverse causality (i.e., that *S. *Typhi detection may be due to the presence of cancer rather than the cause of the cancer), although the consistency of the positive associations between *S. *Typhi and GBC in the meta‐analysis, particularly among the cohort studies, argues against reverse causality. The meta‐analysis was limited by the heterogeneity of the study methods, such as the *S. *Typhi diagnostic methods used.

This study also has a number of strengths. It was conducted in a country with high rates of GBC and a past high incidence of *S. *Typhi in multiple regions. The controls were representative of the population from which the cases arose, reducing the potential for bias between comparison groups, and the extensive epidemiologic data collected allowed for evaluation and control of confounders. In addition, we conducted a large meta‐analysis of over 1000 GBC cases, which allowed us to examine variability by study characteristics. Despite the heterogeneity in study methods, the associations between *S*. Typhi and GBC were consistently positive.

In this study, we found evidence supporting an association between high Vi antibody titer levels and GBC that was consistent with summary estimates from our meta‐analysis of the published literature. While the specific mechanisms involved in *S. *Typhi‐related carcinogenesis may not be clear, the positive association between *S. *Typhi and GBC in the published literature is surprisingly consistent, despite differences in study design. Studies are underway to identify the Vi antibody level cut point to screen for chronic biliary carriers using biotinylated Vi and antigen. In addition, transdisciplinary research between epidemiologists and basic scientists is needed to establish the nature of the association between *S. *Typhi and GBC. Future studies would ideally include both circulating markers of *S. *Typhi exposure and local measures in bile and stool to provide a more complete picture of infection status. Such studies may help inform public health policies regarding *S. *Typhi carriers.

## Conflict of interest

None declared.

## Supporting information


**Table S1**. Association between high titers of IgG antibody to the Vi capsular polysaccharide of *Salmonella enterica* serovar Typhi in gallbladder cancer cases compared to gallstone controls and population‐based controls without gallstones (combined) using various cut‐offs for the definition of high titers.
**Table S2**. Summary of bacteria detected in bile, gallstones, gallbladder tissue, and stool from gallbladder cancer (GBC), gallstone, and population‐based participants in the Shanghai Biliary Tract Cancer Study.

## Appendix

Gallbladder Cancer Chile Working Group (GBCChWG) **Santiago, Chile:** Carmen Gloria Aguayo^8^, Sergio Baez^8^, Alfonso Díaz^4^, Héctor Molina^4^, Carolina Miranda^4^, Claudia Castillo^4^, Andrea Tello^8^. **Concepción, Chile:** Gonzalo Durán^9^, Carolina Paz Delgado^10^, Rodrigo Torres‐Quevedo^9^, Susana Pineda^9^, Tiare de la Barra^9^, Cristian Reyes^9^, Cristina Alegría^9^, Claudia Aguayo^9^. **Temuco, Chile:** Héctor Losada^11,12,13^, Juan Carlos Arraya^11,12,13^, Enrique Bellolio^11,12,13^, Oscar Tapia^11^, Jaime López^5^, Karie Medina^11^, Paulina Barraza^11^, Sandra Catalán^11^, Pía Riquelme^11^, Lorena Órdenes^11^, Raúl Garcés^11^, Claudia Duarte^11^. **USA**: Allan Hildesheim, PhD^1^

^4^Department of Gastroenterology, School of Medicine, Pontificia Universidad Católica de Chile, Santiago, Chile; ^5^Stanford Cancer Institute, Palo Alto, California, USA; ^8^Center for Vaccine Development, University of Maryland School of Medicine, Baltimore, MD USA; ^9^Hospital Dr. Sotero del Río, Santiago, Chile; ^10^Hospital Dr. Guillermo Grant Benavente, Concepción, Chile; ^11^School of Medicine, Universidad de Concepción, Concepción, Chile; ^12^Hospital Dr. Hernan Henríquez Aravena, Temuco, Chile; ^13^Faculty of Medicine, Universidad de La Fontera, Temuco, Chile

## References

[1] Bertran, E. , K. Heise , M. E. Andia , and C. Ferreccio . 2010 Gallbladder cancer: incidence and survival in a high‐risk area of Chile. Int. J. Cancer 127:2446–2454. 20473911 10.1002/ijc.25421

[2] Ferreccio, C . 2011 Salmonella typhi and Gallbladder Cancer in: Bacteria and Cancer. Ed. Khan A. A. Springer, Dordrecht Heidelberg, London, New York.

[3] Gabrielli, M. , S. Hugo , A. Domínguez , S. Baez , A. Venturelli , M. Puga , et al. 2010 Mortality due to gallbladder cancer: retrospective analysis in three Chilean hospitals. Rev. Med. Chil. 138:1357–1364. 21279247

[4] Wernberg, J. , and D. Lucarelli . 2014 Gallbladder Cancer. Surg. Clin. North Amer. 94:343–360. 24679425 10.1016/j.suc.2014.01.009

[5] Lai, C. H. , and W. Y. Lau . 2008 Gallbladder cancer–a comprehensive review. Surgeon 6:101–110. 18488776 10.1016/s1479-666x(08)80073-x

[6] Maringhini, A. , J. A. Moreau , L. J. 3rd Melton , V. S. Hench , A. R. Zinsmeister , and E. P. DiMagno . 1987 Gallstones, gallbladder cancer, and other gastrointestinal malignancies. An epidemiologic study in Rochester, Minnesota. Ann. Intern. Med. 107:30–35. 3592446 10.7326/0003-4819-107-1-30

[7] Axelrod, L. , A. M. Munster , and T. F. O'Brien . 1971 Typhoid cholecystitis and gallbladder carcinoma after interval of 67 years. JAMA 217:83. 5108716

[8] Welton, J. C. , J. S. Marr , and S. M. Friedman . 1979 Association between hepatobiliary cancer and typhoid carrier state. Lancet 1:791–794. 86039 10.1016/s0140-6736(79)91315-1

[9] Mellemgaard, A. , and K. Gaarslev . 1988 Risk of hepatobiliary cancer in carriers of Salmonella typhi. J. Natl Cancer Inst. 80:288. 10.1093/jnci/80.4.288 3351964

[10] Caygill, C. P. G. , M. J. Hill , M. Braddick , and J. C. M. Sharp . 1994 Cancer mortality in chronic typhoid and paratyphoid carriers. Lancet 343:83–84. 7903779 10.1016/s0140-6736(94)90816-8

[11] Ferlay, J. , I. Soerjomataram , M. Ervik , R. Dikshit , S. Eser , C. Mathers , et al. 2013 Cancer Incidence and Mortality Worldwide: IARC CancerBase No. 11 [Internet]. GLOBOCAN 2012 v1.0, Available at http://globocan.iarc.fr, (accessed 12 June 2015). International Agency for Research on Cancer, Lyon, France.

[12] Andia, K. M. , and G. A. Gederlini . 2006 Ferreccio RC [Gallbladder cancer: trend and risk distribution in Chile]. Rev. Med. Chil. 134:565–574. 16802048 10.4067/s0034-98872006000500004

[13] Tsuchiya, Y. , M. Terao , K. Okano , K. Nakamura , M. Oyama , K. Ikegami , et al. 2011 Mutagenicity and mutagens of the red chili pepper as gallbladder cancer risk factor in Chilean women. Asian Pac. J. Cancer Prev. 12:471–476. 21545215

[14] Laval, E. 2007 Ferreccio C [Typhoid fever: rise, peak and fall of an infectious disease in Chile]. Rev. Chilena Infectol. 24:435–440. 18180816

[15] IARC . CANCERMondial. Accessible at http://www-dep.iarc.fr/2009 (accessed 12 June 2015).

[16] Nogueira, L. , C. Foerster , J. Groopman , P. Egner , J. Koshiol , and C. Ferreccio . 2015 Gallbladder Cancer Chile Working G. Association of aflatoxin with gallbladder cancer in Chile. JAMA 313:2075–2077. 26010638 10.1001/jama.2015.4559 PMC7169945

[17] Nagaraja, V. , and D. Eslick . 2014 Systematic review with meta‐analysis: the relationship between chronic Salmonella typhi carrier status and gall‐bladder cancer. Aliment. Pharmacol. Ther. 39:745–750. 24612190 10.1111/apt.12655

[18] Losonsky, G. A. , C. Ferreccio , and K. L. Kotloff . 1987 Development and evaluation of an enzyme‐linked immunosorbent assay for serum Vi antibodies for detection of chronic Salmonella typhi carriers. J. Clin. Microbiol. 25:2266–2269. 3429619 10.1128/jcm.25.12.2266-2269.1987 PMC269467

[19] Wahid, R. , M. F. Pasetti , M. Jr Maciel , J. K. Simon , C. O. Tacket , M. M. Levine , et al. 2011 Oral priming with Salmonella Typhi vaccine strain CVD 909 followed by parenteral boost with the S. Typhi Vi capsular polysaccharide vaccine induces CD27 + IgD‐S. Typhi‐specific IgA and IgG B memory cells in humans. Clin. Immunol. 138:187–200. 21146460 10.1016/j.clim.2010.11.006 PMC3035995

[20] Szu, S. C. , S. Hunt , G. Xie , J. B. Robbins , R. Schneerson , R. K. Gupta , et al. 2013 A human IgG anti‐Vi reference for Salmonella typhi with weight‐based antibody units assigned. Vaccine 31:1970–1974. 23422143 10.1016/j.vaccine.2013.02.006 PMC3839630

[21] Massi, M. N. , T. Shirakawa , A. Gotoh , A. Bishnu , M. Hatta , and M. Kawabata . 2005 Quantitative detection of Salmonella enterica serovar Typhi from blood of suspected typhoid fever patients by real‐time PCR. Int. J. Med. Microbiol. 295:117–120. 15969472 10.1016/j.ijmm.2005.01.003

[22] Fukushima, H. , Y. Tsunomori , and R. Seki . 2003 Duplex real‐time SYBR green PCR assays for detection of 17 species of food‐ or waterborne pathogens in stools. J. Clin. Microbiol. 41:5134–5146. 14605150 10.1128/JCM.41.11.5134-5146.2003 PMC262470

[23] Fernandes‐Monteiro, A. G. , G. F. Trindade , A. M. Yamamura , O. C. Moreira , V. S. de Paula , A. C. Duarte , et al. 2015 New approaches for the standardization and validation of a real‐time qPCR assay using TaqMan probes for quantification of yellow fever virus on clinical samples with high quality parameters. Hum. Vaccin. Immunother. 11:1865–1871. 26011746 10.4161/21645515.2014.990854 PMC4514303

[24] Kuramitsu, M. , K. Okuma , M. Yamagishi , T. Yamochi , S. Firouzi , H. Momose , et al. 2015 Identification of TL‐Om1, an adult T‐cell leukemia (ATL) cell line, as reference material for quantitative PCR for human T‐lymphotropic virus 1. J. Clin. Microbiol. 53:587–596. 25502533 10.1128/JCM.02254-14 PMC4298509

[25] Caygill, C. P. , M. Braddick , M. J. Hill , R. L. Knowles , and J. C. Sharp . 1995 The association between typhoid carriage, typhoid infection and subsequent cancer at a number of sites. Eur. J. Cancer Prev. 4:187–193. 7767246 10.1097/00008469-199504000-00010

[26] Hazrah, P. , K. T. Oahn , M. Tewari , A. K. Pandey , K. Kumar , T. M. Mohapatra , et al. 2004 The frequency of live bacteria in gallstones. HPB (Oxford) 6:28–32. 18333042 10.1080/13651820310025192 PMC2020648

[27] Nath, G. , H. Singh , and V. K. Shukla . 1997 Chronic typhoid carriage and carcinoma of the gallbladder. Eur. J. Cancer Prev. 6:557–559. 9496458 10.1097/00008469-199712000-00011

[28] Pandey, M. , and V. K. Shukla . 2003 Lifestyle, parity, menstrual and reproductive factors and risk of gallbladder cancer. Eur. J. Cancer Prev. 12:269–272. 12883378 10.1097/00008469-200308000-00005

[29] Shukla, V. K. , H. Singh , M. Pandey , S. K. Upadhyay , and G. Nath . 2000 Carcinoma of the gallbladder–is it a sequel of typhoid? Dig. Dis. Sci. 45:900–903. 10795752 10.1023/a:1005564822630

[30] Singh, H. , M. Pandey , and V. K. Shukla . 1996 Salmonella carrier state, chronic bacterial infection and gallbladder carcinogenesis. Eur. J. Cancer Prev. 5:144. 8736080 10.1097/00008469-199604000-00010

[31] Nath, G. , and Y. K. Singh . 2008 Association of carcinoma of the gallbladder with typhoid carriage in a typhoid endemic area using nested PCR. J. Infec. Dev. Ctries. 2:302–307. 19741293 10.3855/jidc.226

[32] Sharma, V. , V. S. Chauhan , G. Nath , A. Kumar , and V. K. Shukla . 2007 Role of bile bacteria in gallbladder carcinoma. Hepatogastroenterology 54:1622–1625. 18019679

[33] Vaishnavi, C. , R. Kochhar , G. Singh , S. Kumar , S. Singh , and K. Singh . 2005 Epidemiology of typhoid carriers among blood donors and patients with biliary, gastrointestinal and other related diseases. Microbiol. Immunol. 49:107–112. 15722595 10.1111/j.1348-0421.2005.tb03709.x

[34] Vaishnavi, C. , S. Singh , R. Kochhar , D. Bhasin , G. Singh , and K. Singh . 2005 Prevalence of Salmonella enterica serovar typhi in bile and stool of patients with biliary diseases and those requiring biliary drainage for other purposes. Jpn. J. Infect. Dis. 58:363–365. 16377868

[35] Tewari, M. , R. R. Mishra , and H. S. Shukla . 2010 Salmonella typhi and gallbladder cancer: report from an endemic region. Hepatobiliary Pancreat. Dis. Int. 9:524–530. 20943463

[36] Andia, M. E. , A. W. Hsing , G. Andreotti , and C. Ferreccio . 2008 Geographic variation of gallbladder cancer mortality and risk factors in Chile: a population‐based ecologic study. Int. J. Cancer 123:1411–1416. 18566990 10.1002/ijc.23662 PMC2864002

[37] Hardy, R. J. , and S. G. Thompson . 1998 Detecting and describing heterogeneity in meta‐analysis. Stat. Med. 17:841–856. 9595615 10.1002/(sici)1097-0258(19980430)17:8<841::aid-sim781>3.0.co;2-d

[38] Sterne, J. A. , D. Gavaghan , and M. Egger . 2000 Publication and related bias in meta‐analysis: power of statistical tests and prevalence in the literature. J. Clin. Epidemiol. 53:1119–1129. 11106885 10.1016/s0895-4356(00)00242-0

[39] Begg, C. B. , and M. Mazumdar . 1994 Operating characteristics of a rank correlation test for publication bias. Biometrics 50:1088–1101. 7786990

[40] Egger, M. , G. Davey Smith , M. Schneider , and C. Minder . 1997 Bias in meta‐analysis detected by a simple, graphical test. BMJ 315:629–634. 9310563 10.1136/bmj.315.7109.629 PMC2127453

[41] Csendes, A. , M. Becerra , P. Burdiles , I. Demian , K. Bancalari , and P. Csendes . 1994 Bacteriological studies of bile from the gallbladder in patients with carcinoma of the gallbladder, cholelithiasis, common bile duct stones and no gallstones disease. Eur. J. Surg. 160:363–367. 7948355

[42] Serra, I. , M. Yamamoto , A. Calvo , G. Cavada , S. Baez , K. Endoh , et al. 2002 Association of chili pepper consumption, low socioeconomic status and longstanding gallstones with gallbladder cancer in a Chilean population. Int. J. Cancer 102:407–411. 12402311 10.1002/ijc.10716

[43] Dutta, U. , P. K. Garg , R. Kumar , and R. K. Tandon . 2000 Typhoid carriers among patients with gallstones are at increased risk for carcinoma of the gallbladder. Am J. Gastroenterol. 95:784–787. 10710075 10.1111/j.1572-0241.2000.01860.x

[44] Roa, I. , J. C. Araya , M. Villaseca , J. Roa , X. de Aretxabala , and G. Ibacache . 1999 Gallbladder cancer in a high risk area: morphological features and spread patterns. Hepatogastroenterology 46:1540–1546. 10430291

[45] Strom, B. L. , R. D. Soloway , J. L. Rios‐Dalenz , H. A. Rodriguez‐Martinez , S. L. West , J. L. Kinman , et al. 1995 Risk factors for gallbladder cancer. An international collaborative case‐control study. Cancer 76:1747–1756. 8625043 10.1002/1097-0142(19951115)76:10<1747::aid-cncr2820761011>3.0.co;2-l

[46] Capoor, M. R. , D. Nair , Rajni , G. Khanna , S. V. Krishna , M. S. Chintamani , et al. 2008 Microflora of bile aspirates in patients with acute cholecystitis with or without cholelithiasis: a tropical experience. Braz J. Infect. Dis. 12:222–225. 18839486 10.1590/s1413-86702008000300012

[47] Safaeian, M. , Y. T. Gao , L. C. Sakoda , S. M. Quraishi , A. Rashid , B. S. Wang , et al. 2011 Chronic typhoid infection and the risk of biliary tract cancer and stones in Shanghai, China. Infect. Agent Cancer 6:6. 21535882 10.1186/1750-9378-6-6 PMC3110129

[48] Koshiol J, Wozniak A , Cook P , Adaniel C , Miquel JF , Acevedo J , Hsing A , et al., Group. GCCW . Chronic Salmonella Typhi carriage and gallbladder cancer: a case control and meta‐analysis In Preparation. Cancer Med.

[49] Yagyu, K. , Y. Lin , Y. Obata , S. Kikuchi , T. Ishibashi , M. Kurosawa , et al. , Group JS . 2004 Bowel movement frequency, medical history and the risk of gallbladder cancer death: a cohort study in Japan. Cancer Sci. 95:674–678. 15298731 10.1111/j.1349-7006.2004.tb03328.x PMC11158605

[50] Crawford, R. W. , D. L. Gibson , W. W. Kay , and J. S. Gunn . 2008 Identification of a bile‐induced exopolysaccharide required for Salmonella biofilm formation on gallstone surfaces. Infect. Immun. 76:5341–5349. 18794278 10.1128/IAI.00786-08 PMC2573354

[51] Csendes, A. 1994 Cholecystectomy in young women. Rev. Med. Chil. 122:1086. 7597342

[52] Ristori, C. , H. Rodriguez , P. Vicent , C. Ferreccio , J. Garcia , H. Lobos , et al. 1982 Persistence of the Salmonella typhi‐paratyphi carrier state after gallbladder removal. Bull. Pan Am. Health Organ. 16:361–366. 7165819

[53] Daniels, E. M. , R. Schneerson , W. M. Egan , S. C. Szu , and J. B. Robbins . 1989 Characterization of the Salmonella paratyphi C Vi polysaccharide. Infect. Immun. 57:3159–3164. 2506132 10.1128/iai.57.10.3159-3164.1989 PMC260784

[54] Raffatellu, M. , D. Chessa , R. P. Wilson , C. Tukel , M. Akcelik , and A. J. Baumler . 2006 Capsule‐mediated immune evasion: a new hypothesis explaining aspects of typhoid fever pathogenesis. Infect. Immun. 74:19–27. 16368953 10.1128/IAI.74.1.19-27.2006 PMC1346610

[55] Raffatellu, M. , R. L. Santos , D. Chessa , R. P. Wilson , S. E. Winter , C. A. Rossetti , et al. 2007 The capsule encoding the viaB locus reduces interleukin‐17 expression and mucosal innate responses in the bovine intestinal mucosa during infection with Salmonella enterica serotype Typhi. Infect. Immun. 75:4342–4350. 17591794 10.1128/IAI.01571-06 PMC1951168

[56] Keestra‐Gounder, A. M. , R. M. Tsolis , and A. J. Baumler . 2015 Now you see me, now you don't: the interaction of Salmonella with innate immune receptors. Nat. Rev. Microbiol. 13:206–216. 25749454 10.1038/nrmicro3428

[57] Diez‐Garcia, M. , R. Capita , and C. Alonso‐Calleja . 2012 Influence of serotype on the growth kinetics and the ability to form biofilms of Salmonella isolates from poultry. Food Microbiol. 31:173–180. 22608221 10.1016/j.fm.2012.03.012

[58] Crawford, R. W. , and R. Rosales‐Reyes . 2010 Ramirez‐Aguilar Mde L, Chapa‐Azuela O, Alpuche‐Aranda C, Gunn JS. Gallstones play a significant role in Salmonella spp. gallbladder colonization and carriage. Proc. Natl. Acad. Sci. U. S. A. 107:4353–4358. 20176950 10.1073/pnas.1000862107 PMC2840110

[59] Nath, G. , A. K. Gulati , and V. K. Shukla . 2010 Role of bacteria in carcinogenesis, with special reference to carcinoma of the gallbladder. World J. Gastroenterol. 16:5395–5404. 21086555 10.3748/wjg.v16.i43.5395 PMC2988230

[60] Akhmedov, D. R . 1994 The status of the blood antioxidant system in a chronic typhoid bacterial carrier state. Zh. Mikrobiol. Epidemiol. Immunobiol. 1:91–95. 8184623

[61] Scanu, T. , R. M. Spaapen , J. M. Bakker , C. B. Pratap , L. E. Wu , I. Hofland , et al. 2015 Salmonella Manipulation of Host Signaling Pathways Provokes Cellular Transformation Associated with Gallbladder Carcinoma. Cell Host Microbe 17:763–774. 26028364 10.1016/j.chom.2015.05.002

[62] Roa, I. , G. Ibacache , J. Carvallo , A. Melo , J. Araya , X. De Aretxabala , et al. 1999 Figueroa C [Microbiological study of gallbladder bile in a high risk zone for gallbladder cancer]. Rev. Med. Chil. 127:1049–1055. 10752267

