# Supplementary material for: Salmonella enterica serovar Typhi and gallbladder cancer: a case–control study and meta‐analysis
Source: Cancer Med. 2016 Oct 11;5(11):3310–235. doi: 10.1002/cam4.915 (PMC5119987; doi:10.1002/cam4.915)
Supplement: Supplementary file 1 — Table S1. Association between high titers of IgG antibody to the Vi capsular polysaccharide of Salmonella enterica serovar Typhi in gallbladder cancer cases compared to gallstone controls and population‐based controls without gallstones (combined) using various cut‐offs for the definition of high titers. Table S2. Summary of bacteria detected in bile, gallstones, gallbladder tissue, and stool from gallbladder cancer (GBC), gallstone, and population‐based participants in the Shanghai Biliary Tract Cancer Study. [file CAM4-5-3310-s001.doc]

Supplemental Table 1. Association between high titers of IgG antibody to the Vi capsular polysaccharide of *Salmonella enterica* serovar Typhi in gallbladder cancer cases compared to gallstone controls and population-based controls without gallstones (combined) using various cut-offs for the definition of high titers.

| **Cut-off for high Vi antibody levels** | **OR (95% CI)** | |
| --- | --- | --- |
|  | **Crude** | **Adjusted*** |
| ≥0.2 µg/ml | 2.2 (0.7-7.4) | 2.0 (0.5-7.3) |
| ≥0.3 µg/ml | 4.6 (1.1-19.5) | 4.0 (0.9-18.1) |
| ≥0.5 µg/ml | 2.9 (0.6-13.6) | 2.4 (0.5-12.1) |
| ≥1.0 µg/ml | 2.1 (0.4-11.0) | 1.7 (0.3-9.3) |
| ≥1.5 µg/ml | 2.1 (0.3-15.4) | 1.9 (0.2-15.5) |

*Adjusted for gender and education

Supplemental Table 2. Summary of bacteria detected in bile, gallstones, gallbladder tissue, and stool from gallbladder cancer (GBC), gallstone, and population-based participants in the Shanghai Biliary Tract Cancer Study.

| **Participant type** | **Biospecimen** | **Assay** | **Bacteria** |
| --- | --- | --- | --- |
| GBC | Tissue | PCR | *Salmonella* species |
| GBC | Stool | Culture | *Citrobacter braakii; Providencia rettgeri* |
| GBC | Stool | Culture | *Escherichia coli; Proteus mirabilis* |
| GBC | Gallstones | Culture | *Kytococcus* |
| GBC | Bile Tissue | Culture Culture | *Enterococcus gallinarum Pseudomonas species; Enterococcus gallinarum* |
| Gallstone control | Bile | PCR | *Salmonella species* |
| Gallstone control | Gallstones Tissue | Culture Culture | *Escherichia coli Escherichia coli* |
| Gallstone control | Gallstones Tissue | Culture Culture | *Escherichia coli Escherichia coli* |
| Gallstone control | Gallstones | Culture | *Enterococcus avium* |
| Gallstone control | Gallstones | Culture | *Pseudomonas aeruginosa* |
| Gallstone control | Gallstones | Culture | *Enterococcus faecium* |
| Gallstone control | Gallstones Tissue | Culture Culture | *Citrobacter freundii; Klebsiella oxytoca Enterococcus casseliflavus; Aeromonas caviae; Klebsiella oxytoca* |
| Gallstone control | Tissue | Culture | *Escherichia coli* |
